# Utilizing Incentivized Economic Experiments to Test for Social Skills Acquisition Through Physical Education: Study Protocol of the *Movigen* Project

**DOI:** 10.3389/fspor.2021.587764

**Published:** 2021-05-03

**Authors:** André Haas, Rita Wittelsberger, Hagen Wäsche, Alexander Woll, Petra Nieken

**Affiliations:** ^1^Institute of Sports and Sports Science, Karlsruhe Institute of Technology, Karlsruhe, Germany; ^2^SRH University of Applied Health Sciences, Karlsruhe, Germany; ^3^Institute of Management, Karlsruhe Institute of Technology, Karlsruhe, Germany

**Keywords:** physical education, intervention, economic experiment, social skills, spillover, randomized controlled trial

## Abstract

Besides cognitive skills, non-cognitive skills—social skills in particular—are crucial for outcomes in various domains of life. The present work describes the design of the *Movigen* project, an intervention study with children aged 10–13 years using enhanced physical education lessons to foster social skills in a playful way. Utilizing a novel methodological approach various incentivized economic experiments were applied to test for spillover effects of the intervention on social skills. At three points during the course of the study individuals participated in a series of incentivized economic experiments to elicit economic preferences and personality traits. Additional information about physical activity and free time activities, different psychometric scales, and family background were elicited with questionnaires. Furthermore, a subset of individuals was equipped with accelerometers for 7 days to validate the answers on physical activity in the questionnaire. The data set comprises a treatment group which received enhanced physical education lessons and a control group which received regular physical education lessons at school. The comparison of individuals' decision in the economic experiments between both groups allows to study the impact of our intervention on social skills.

## Introduction

Success in various domains of life, e.g., at school or at the workplace, does not only depend on cognitive skills, but also on non-cognitive skills. In particular, inputs from both realms are frequently necessary to attain desirable results (Borghans et al., 2008). Whereas, cognitive skills refer to attributes which can be measured in terms of scores achieved in standardized tests, non-cognitive skills are more difficult to capture (Heckman and Kautz, 2012; Heckman et al., 2019). One reason is the difficulty to define the construct precisely in the first place (Duckworth and Yeager, 2015). A comprehensive overview on aspects which are comprised by the term non-cognitive skills can be found in, e.g., Farrington et al. (2012) and Gutman and Schoon (2013). The focus of the presented study design is on social skills which are a subset of non-cognitive skills and social competencies, respectively. In particular, social skills cover a set of behavioral facets which are a crucial determinant for effective interaction with other people. Notable instances of social skills are cooperation, interpersonal skills, empathy, assertion, and responsibility (Farrington et al., 2012; Gutman and Schoon, 2013).

Whereas, requirements at the workplace increasingly emphasize the importance of non-cognitive skills (Deming, 2017; Bode et al., 2019; see also Weidmann and Deming, 2020), employers are at the same time concerned with a lack of social skills in applicants which is detrimental for employability (European Commission, 2016, 2017). One approach to enhance social skills are intervention programs. As non-cognitive skills are especially malleable at young ages, these programs are often directed toward children and adolescents (see Kautz et al., 2014, for an overview). The aim of the present study is to convey social skills in a playful way during purposefully designed physical education lessons (see Woll et al., 2018, for a description of the curriculum).

Besides positive effects on health (Warburton et al., 2006; Reiner et al., 2013; Pedersen and Saltin, 2015), physical activity and sports participation are often conjectured to improve outcomes in other domains of life as well. One explanation is that being involved in an organizational structure, e.g., in a sports club, yields spillover effects which foster the development of social skills as individuals have to get along with other individuals from diverse backgrounds to pursue common goals (Pfeifer and Cornelißen, 2010; Cabane and Clark, 2015; Felfe et al., 2016). Causal evidence on the impact of physical activity and sports participation on the development of social skills is, however, scarce (Pawlowski et al., 2018). Yet, the meta-study by Schüller and Demetriou (2018) finds positive effects of sports-related interventions at schools using randomized controlled trials on social skills of children and adolescents for the vast majority of the underlying studies.

For the purpose of this study we developed a novel methodological approach. To investigate the impact of our intervention and to assess potential spillover effects, individuals' social skills were measured at three different times. This is done by a series of well-established economic experiments. These experiments employ an incentive-compatible reward structure based on the individuals' decisions (and decisions of their class mates in some instances) in various economic environments which induce participants to reveal their true preferences. Whereas, responses on batteries of self-reported questions may be subject to individual biases due to different readings of the underlying scales, the current approach uses an objective scale provided all participants value monetary payoffs in the same way (Golsteyn and Schildberg-Hörisch, 2017; see also Heckman et al., 2019). To our best knowledge, there are no previous studies using a comparable approach to test for spillover effects of a sports-related intervention on other domains of life utilizing incentivized economic experiments.

## Outline of the Study

### Participants and Allocation

Students at four different upper secondary schools in Karlsruhe (Germany) participated in the study. In total, students attending eight different classes of the sixth grade participated in the study. To assess the impact of the intervention on students' personal and social skills, a randomized controlled trial employing a treatment and a control group was necessary. This allows to disentangle the effect of the intervention from other influences outside the control of this study as the latter can be assumed to affect students in the treatment and the control group in the same way. At each school, one class was, therefore, randomly allocated to the treatment and control group, respectively. Due to fairness concerns, we offered the classes of the control group to receive enhanced physical education lessons as conducted during the intervention in the treatment group after the end of the study.

To be eligible for participation, parental consent in written form was mandatory. Therefore, we distributed letters inviting their children to participate in a scientific study during regular lessons to the parents via the respective schools. No information about the purpose of the study was disclosed, neither to the parents nor to the teachers. Furthermore, the superintendent of the local school district (ref.-no. 71 c2-6499.25) and the Board of Ethics of the Karlsruhe Institute of Technology approved the study.

One hundred and Sixty-six of the 197 students attending the classes under study (84.3%) returned the consent form and were, thus, allowed to participate in the study. Due to absence in one or more parts of the study, data are not complete for some individuals. Hence, the final data set contains observations on 111 individuals aged 10–13 years (mean: 11.7 years, standard deviation: 0.44 years). Table 1 provides an overview on the distribution female and male individuals in the respective groups who participated in all parts of the study.

**Table 1:** Number of individuals by group and gender.

	**Female**	**Male**	**Total**
Control	27 (44%)	35 (56%)	62
Treatment	25 (51%)	24 (49%)	49
	52 (47%)	59 (53%)	111

### Set-Up

**Figure 1:**
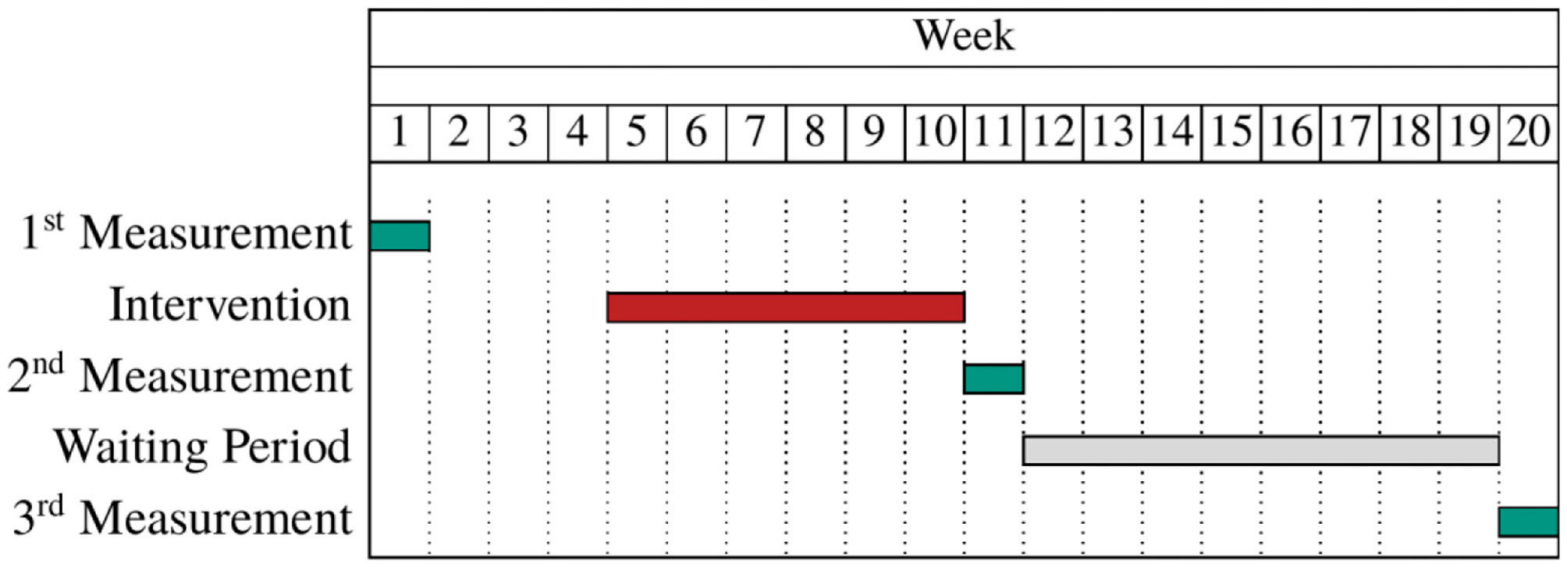
Study set-up.

Figure 1 presents an outline of the study. Individuals participated in three measurements to elicit social skills in a series of different economic experiments during the course of the study. Repeated measurements were necessary to study both, individuals' immediate responses to the intervention as well as long-term effects. This information is complemented with data from questionnaires on free time activities (DIW/SOEP, 2015c) and engagement in physical activities (Schmidt et al., 2016) as well as several psychometric scales such as the Big Five inventory (Weinhardt and Schupp, 2011), social self-efficacy, self-efficacy of working in a team, perspective-taking (Jerusalem et al., 2009), rivalry (Eder, 1998; see also Kunter et al., 2002), and understanding of democracy (Abs et al., 2007). These psychometric scales were included to control for impacts of the intervention on individuals' personalities which has been proposed in the literature (e.g., Schmidt and Conzelmann, 2011). Questionnaires for individuals participating in the study were distributed after each measurement and were answered at home. Parents also answered a questionnaire after the first measurement to provide additional information about their children, characteristics of the household, and their own educational background (based upon DIW/SOEP, 2015a,b). At one school, individuals in the treatment and control group wore accelerometers for 7 days after the first measurement to collect data about their overall physical activity in order to validate the corresponding answers in the questionnaire.

### Intervention

Individuals of the treatment group participated in an intervention for a period of 6 weeks between the first and second measurement. The intervention took place during regular physical education lessons and had been designed to enrich the curriculum with a novel concept which conveys social skills in a playful way. Students participated in one lesson per week. Topics of the intervention were cooperation and willingness to work in a team, formal and informal fairness, self-assessment, and conflict resolution with one specific topic being covered in each lesson. To put individuals in situations which require them to actively work on solutions, the main part of each lesson consisted of physical activities related to the respective topic of a lesson. To foster the transfer of social skills to situations in their everyday lives, students were encouraged to share their experiences and impressions during the first and final 10 min of each lesson. Note that the social skills elicited in the incentivized economic experiments and the topics covered in the intervention are only loosely related. This is a deliberate decision in the design of the present study since it is not its purpose to teach particular behavioral patterns. In fact, the goal is to provide a playful environment which encourages individuals to develop skills which enable them to solve challenges in various domains of life on their own.

Lessons were conducted by university students of sports science and physical education who had experience in instructing children and adolescents in sports classes. They had received additional training prior to conducting the intervention, but were otherwise not aware of the purpose of this study. Woll et al. (2018) contains a description of the intervention in more detail. For a period of 6 weeks, classes which had been assigned to the treatment group received one lesson per week. Each lesson had been outlined for a slot of 80 min:

- The first 15 min (approximately) were used for a warm-up to prevent injuries.
- After that, the topic of the specific lesson was introduced to the class (approximately 8 min). Students were asked to contribute own experiences related to the respective topic to establish a common understanding.
- The main part of the lesson (approximately 50 min) addressed the respective topic utilizing a playful approach. Therefore, the topic of a lesson was presented in a sports-related context, e.g., in terms of an additional rule imposed on a game which required the students' consideration. To achieve a transmission beyond the setting of the intervention, students were encouraged to actively develop a way to deal with this kind of constraint within their classes. It is important to note that—although the intervention is designed to supplement regular physical education lessons—the goal of this study is not to promote physical activity in the first place. Rather, the specific setting of physical education lessons provides an environment which allows to pursue a playful approach to foster social skills as students receive instantaneous feedback from their peers which enables them to adapt their behavior accordingly.
- Each lesson was concluded by a short period of reflection (approximately 7 min) in which students had the opportunity to share their impressions and take private notes in a diary which had been handed out upfront.

Moreover, the design of the intervention took particular consideration to establish an environment which promotes the acquisition of new behaviors and their transmission beyond the setting of the intervention. As one element of the intervention was the development of own solution concepts by students, instructors were told to monitor their classes during the main part of each lesson but not to intervene unless to prevent injuries or turmoil. Feedback was another important parameter. Students were, therefore, instructed to provide each other non-judgmental feedback based on their behavior in specific situations and suggestions for future improvements—or appreciation.

## Experimental Design

As mentioned above, a wide range of individual behaviors is summarized under the umbrella term social skills which are not trivial to measure. Whereas, this term is not used uniformly across different disciplines, we take reference to the understanding proposed in behavioral economics and economic psychology, respectively, where social skills comprise—among others—economic preferences and other determinants which are crucial for various life outcomes (Humphreys and Kosse, 2017). This enables us to build on a range of incentivized tasks which allow to draw inferences on underlying preference parameters (Ertac, 2020). To elicit individual preferences and traits in different economic environments we used a series of economic experiments. To establish an incentive-compatible setting for the elicitation of social skills, one experiment was selected for payout at the end of each measurement (see section Procedures).

Table 2 provides an overview of preferences and traits which are of particular interest in context of the present study as well as the corresponding economic experiments used for elicitation and the outcome variables of interest. Moreover, this section provides an overview on predictions of individuals' behavior in the economic experiments. A comprehensive overview on children's decisions in different economic experiments is provided by Sutter et al. (2019).

**Table 2:** Overview of social skills measured by different economic experiments.

**Social skills**	**Economic experiments**	**Outcome variables**
Altruism	Ultimatum game (sender)	Amount passed to the receiver (ratio scale)
	Dictator game	Amount passed to the receiver (ratio scale)
Negative reciprocity	Ultimatum game (receiver)	Negatively reciprocal if positive offer is rejected (binary scale)
Cooperation	Public good game	Amount contributed to the collective account (ratio scale)
	Prisoner's dilemma	Three types: (i) always cooperate; (ii) never cooperate; (iii) conditionally cooperate (categorical scale)
Time preferences	Piggy bank	Patient if delayed gratification is chosen (binary scale)
Honesty	Mind game	Honesty on *aggregate* level if share of matching numbers does not exceed 1/6 (binary scale)
Risk preferences	Lottery	Three types: (i) risk-neutral; (ii) risk-averse; (iii) risk-seeking (categorical scale)
Competitiveness	Self-selection into payment scheme^*^	Competitive if tournament scheme is chosen (binary scale)
Overconfidence	Estimation of relative performance^*^	Overconfident if result falls into a lower tercile than estimated (binary scale)
	Selection of task difficulty^*^	Overconfident if threshold of chosen difficulty level is not achieved (binary scale)

* *Operationalized using the encryption task*.

### Ultimatum and Dictator Game

The ultimatum game (Güth et al., 1982; Güth and Kocher, 2014) depicts a bargaining situation between two individuals, sender and receiver. To elicit a complete strategy profile for each individual, we employ the strategy method (Selten, 1967). In the first stage, all individuals make their individual decision in the role of the sender. They are endowed with 600 tokens each and have to decide which fraction *x* ϵ {0, 120, 300, 480, 600} they pass to the receiver who is one randomly selected individual of the same class. The remaining 600 – *x* tokens are kept by the sender. In the second stage, all individuals decide individually for each potential offer *x* a sender can make whether or not they want to accept this offer. If this experiment is chosen for payment, two individuals are randomly matched who are assigned the role of the sender and receiver, respectively. If the receiver chose “accept” for the respective sender's offer, the receiver receives *x* tokens and the sender receives 600 – *x* tokens. If the receiver chose “not accept,” both individuals receive a payoff of zero tokens.

The ultimatum game employs a sequential structure. Thus, backward induction is used to derive predictions for both, the sender and the receiver. In the second stage, the receiver has to decide for each potential offer x the sender can make whether she wants to accept the offer or not. A selfish receiver who maximizes her own payoff accepts any positive offer. Thus, the sender offers the smallest positive amount, i.e., 120 tokens, while keeping the remaining 480 tokens for herself—and the receiver accepts. The dictator game, in contrast, does not incorporate any decision by the receiver. Hence, the sender will pass the smallest possible amount, i.e., 0 tokens, to the receiver and keep the entire endowment of 600 tokens for herself (Hoffman et al., 2008).

The dictator game is a modification of the ultimatum game (Forsythe et al., 1994). Again, individuals are endowed with 600 tokens. They decide which fraction *x* ϵ {0, 120, 300, 480, 600} they pass to the receiver while keeping the remaining 600 – *x* tokens. Unlike in the ultimatum game, the receiver cannot reject the allocation of the sender. Moreover, the receiver in this setting is not another individual of the same class. Instead, tokens sent to the receiver are donated to a charity which supports children and their families in developing countries if this experiment is selected for payment.

In contrast to standard theoretical predictions, offers between 40 and 50% of the initial budget by senders are commonly observed in ultimatum games. Receivers regularly accept these offers. As offers decline, rejection rates increase—in particular for offers below 20% of the initial budget which are rejected in most of the cases (Camerer, 2003). With a share of about 30% of the initial budget which is passed to the recipient in the dictator game the amount ranges below the frequently observed offers in the ultimatum game. In particular, a substantial fraction of more than one-third of dictators decide to keep the entire budget (Engel, 2011). On the majority, fifth-graders aged 10–11 years in the study of Angerer et al. (2015) pass one to three tokens of an endowment of six tokens to a charity. Only 13% of the individuals keep their entire endowments and a fraction of <10% of the individuals passes more than half of their endowments to the charity.

A positive amount passed to the receiver in the ultimatum and dictator game reflects the sender's unconditional and conditional altruism, respectively. More precisely, the amount passed to the receiver is a measure for the extent of the sender's unconditional and conditional altruism. A receiver in the ultimatum game who rejects a positive amount offered by the sender is demonstrates negatively reciprocal behavior.

### Prisoner's Dilemma and Public Good Game

**Figure 2:**
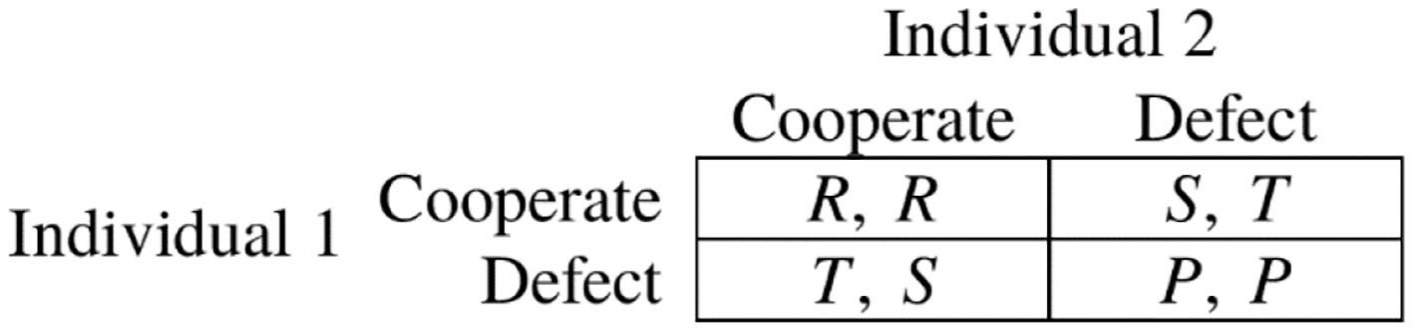
Prisoner's dilemma. *T* (Temptation) > *R* (Reward) > *P* (Punishment) > *S* (Sucker's payoff) and 2*R* > *T* + *S*.

The prisoner's dilemma is a two-player experiment which constitutes a social dilemma (Roth, 1995). Each individual can choose between two strategies, “Cooperate” and “Defect.” The payoff matrix is depicted in Figure 2. To elicit a complete strategy profile for each individual, we use the strategy method. In the first stage, each individual decides independently whether she wants to cooperate or defect. In the second stage, individuals face the same decision contingent on the other individual's possible decision to (i) cooperate and (ii) defect. If the experiment is selected for payment, two individuals of a class are randomly matched. Within each group, one individual is randomly selected whose independent decision is relevant for payoff. For the other player, the corresponding dependent decision is evaluated. Both individuals' payoffs are denoted by the values of the resulting cell of the payoff matrix.

The public good game is closely related to the prisoner's dilemma as it constitutes a social dilemma in groups with two or more individuals (Ledyard, 1995; Roth, 1995; Cipriani et al., 2013). Individuals are endowed with 120 tokens and form a group with three other randomly selected individuals of the same class. They can allocate their endowment to a private or a public account in intervals of 24 tokens. If the experiment is selected for payment, the number of tokens allocated to the collective account by all individuals of the group is totaled. Each individual receives a return of 1 token for every token contributed to the collective account—no matter by whom. Additionally, each individual receives a return of 2 tokens for every token allocated to her private account. Thus, the payoff π_*i*_ of individual *i* is given by

$$\begin{array}{l} \pi_{i}=2\left(120-c_{i}\right)+\left(c_{1}+c_{2}+c_{3}+c_{4}\right), \end{array}$$

where *c*_*i*_ ϵ {0, 120, 300, 480, 600} is individual *i*'s contribution to the collective account as multiple of 24 tokens. The marginal per-capita return (MPCR) is 0.5, i.e., every token contributed to the collective account by an individual herself yields a return which is half the quantum of the return from a contribution of one token to her private account.

Using the strategy method in the prisoner's dilemma allows to identify three types of contributors (similar to Fischbacher et al., 2001). Individuals who always cooperate or defect, irrespectively of the other individual's decision (“always cooperate” and “never cooperate,” respectively) and “conditional cooperators” who cooperate if the other individual does so and vice versa if the other individual defects. In the public good game, the amount of tokens contributed to the collective account indicates the individual level of cooperativeness.

### Lottery

The lottery consists of two experiments (conceptually similar to Deckers et al., 2017; see also Castillo et al., 2018). In each experiment, individuals choose between two envelopes. In the first experiment, envelope A contains six green cards and envelope B contains three red cards and three blue cards. If this experiment is selected for payment, a card is drawn from each envelope A and B. An individual who chose envelope A receives 200 tokens if a green card is drawn (probability: 100%). An individual who chose envelope B receives 600 tokens if a red card is drawn (probability: 50%) and zero tokens if a blue card is drawn (probability: 50%). The second experiment is identical to the first except the names of the envelopes and the colors of the cards: Envelope C contains six orange cards and envelope D contains three purple cards and three yellow cards. If this experiment is selected for payment, an individual who chose envelope C receives 400 tokens if an orange card is drawn (probability: 100%). An individual who chose envelope D receives 600 tokens if a purple card is drawn (probability: 50%) and zero tokens if a yellow card is drawn (probability: 50%).

Envelopes A and C represent degenerate lotteries with a sure payoff of 200 tokens and 400 tokens, respectively. Envelopes B and D represent the same non-degenerate lottery *L* = (0.5°600, 0.5°0) with an expected payoff E[*L*] = 300 tokens. An individual who chooses the lottery (envelope B) in the first experiment (E[*L*] > 200) and the sure option (envelope C) in the second experiment (400 > E[*L*]) is labeled risk-neutral. Similarly, an individual who always chooses the sure payoff (envelopes A and C) or the lottery (envelopes B and D) is labeled risk-averse or risk-seeking, respectively. In a comparable setting, Deckers et al. (2017) identify about 44% of children aged 7–9 years in their sample as risk-neutral. While this is independent of their socio-economic status, children from households with high socio-economic status are less often risk-seeking and more often risk-averse than their counterparts from households with low socio-economic status.

### Encryption Task

In a real-effort task adopted from Erkal et al. (2011) individuals have to replace letters of a given word. The difficulty of the task increases in the number of iterations which are required for the encryption. For the one-fold encryption, the letters of the original word have to be replaced once by another letter from a given table. The two-fold variant requires an additional step, i.e., the letters of the original word have to be replaced in two subsequent steps according to two different given tables, and so forth. The number of correctly encrypted letters in a 2-min working period is the outcome variable of interest. The task is employed in three different experiments with a specific payoff structure each:

1. At the beginning of this experiment, individuals can familiarize with the task during a trial period of 30 s in which they are asked to apply a two-fold encryption on a word with five letters. The main part also requires two-fold encryption. Before they start working, individuals give an estimate about their performance in the main part relative to the performance of the other individuals of their class, i.e., they indicate whether they expect themselves to be among the top, middle, or lower third (Almås et al., 2016). If this experiment is selected for payment, a correct estimation yields 300 tokens while an incorrect estimation results in a payoff of zero tokens. Additionally, individuals receive 3 tokens for each correctly encrypted letter with a cap at 300 tokens.
2. In this setting, individuals work again on the two-fold encryption with different replacement tables. Before they start working, individuals can choose whether they prefer a piece-rate or a payment based on a tournament scheme (Niederle and Vesterlund, 2007; Booth and Nolen, 2012; Samak, 2013; Almås et al., 2016). The piece-rate is 3 tokens for every correctly encrypted letter with a cap at 300 tokens, whereas the tournament scheme comprises a loser prize of zero tokens and a winner prize of 600 tokens. An individual who chose the tournament scheme receives the winner prize if this experiment is selected for payoff if she obtained a higher output than another randomly chosen individual of the same class. If the individual under question obtained a lower output, she receives the loser prize. Ties are broken randomly.
3. In this variant, individuals can choose between three different levels of task difficulty (similar to Falk et al., 2015)^1^ which require one-, two-, and three-fold encryption, respectively. If this experiment is selected for payment, the remuneration depends on the level of task difficulty according to Table 3. For each level of task difficulty, the payoff is contingent on a threshold which specifies a minimum number of letters which have to be encrypted correctly. At the same time, the maximum amount of tokens which can be achieved is capped for each level of task difficulty.

**Table 3:** Payoff by level of task difficulty in the encryption task.

**Task difficulty**	**Piece-rate**	**Threshold**	**Cap**
One-fold encryption	1 token	5 letters	100 tokens
Two-fold encryption	3 tokens	15 letters	300 tokens
Three-fold encryption	10 tokens	35 letters	1,000 tokens

The specific payment schemes in the encryption task relate to different personality traits. The first and the third variant are concerned with relative and absolute self-assessment, respectively. These personality traits refer to individuals' ability to estimate their own performance accurately in comparison to their peers or in absolute terms. Individuals who expect themselves to perform better in the relative ranking of their class than they actually do are deemed overconfident. Similarly, individuals who do not achieve the minimum number of correctly encrypted letters required under the level of task difficulty of their choice fall into the same category. Falk et al.^1^ find 37% of the children in their sample with an average age of 8 years to be overconfident prior to their intervention.

The choice between two compensation schemes either based on an individual's absolute performance or on her relative performance compared to another randomly selected individual of the same class is an indicator for individuals' competitiveness. More precisely, competitive individuals choose the tournament-based compensation scheme. The seminal paper by Niederle and Vesterlund (2007) reports that male individuals are more competitive than females as three quarter of men and one-third of women prefer the tournament scheme. This gender gap also occurs among children, although its magnitude is less pronounced (e.g., Almås et al., 2016).

### Mind Game

In the mind game (Jiang, 2013; see also Kajackaite and Gneezy, 2017; Abeler et al., 2019), individuals are first asked to imagine an integer number between one and six. After that, they receive an envelope each. The envelope contains a set six cards with one number from one to six printed on each card. Individuals are then asked to draw one card from the envelope and to indicate whether the number on the drawn card matches the integer they had imagined before. If this experiment is selected for payment, individuals who reported a match between the imagined and the drawn number receive 600 tokens and zero tokens otherwise.

The mind game addresses dishonesty. The experiment deliberately does not allow to derive conclusions whether an individual who reported matching numbers was actually dishonest or not. On the aggregate level, however, the fraction of individuals who report matching numbers is expected to be 1/6 in a sufficiently large sample. Hence, a share of matching numbers reported which exceeds 1/6 indicates dishonest behavior on an aggregate level. In a similar setting in which children aged 5–15 years in Italy received a payment conditional on the outcome of a fair coin toss, about 85% of the individuals reported an outcome which entitled to receive the payment. This fraction was substantially higher than the theoretical share of 50% (Bucciol and Piovesan, 2011).

### Piggy Bank

This experiment offers the choice between two alternatives which differ in the amount of the payoff and its date (Bauer et al., 2014; Angerer et al., 2015; Deckers et al., 2017). If the experiment is selected for payoff, individuals who chose the first alternative receive 300 tokens on the same day, whereas individuals who opted for the second alternative receive 600 tokens with a delay of 1 week.

The decision in the piggy bank experiment measures time preferences. Individuals who trade off an earlier gratification for a higher payoff exhibit a low discounting rate and are deemed patient. In a similar setting, 63% of the children between 4 and 12 years (average age about 8 years) who participated in a study in the Czech Republic chose the higher payoff with a delay of 1 week (Bauer et al., 2014).

### Procedures

As described in section Set-Up, individuals participated at three times during the course of the study in a measurement using a series of standard economic experiments to elicit different social skills. Measurements took place in classrooms during regular lessons and were conducted by students of different fields of study who were pursuing a university degree which qualifies for teaching at upper secondary schools; they were not aware of the purpose of this study.

To identify individuals across different stages of the study while maintaining anonymity, they received a card with their ID number at the beginning of each measurement from their teacher. Individuals' identities were not disclosed to the experimenters. During the measurements, individuals were allocated in the classroom such that they were able to make their decisions independently and without being disturbed. General instructions were read aloud before the first experiment. After clarifying questions about the general procedure had been answered, individuals received the answer sheet for the first experiment. The experimenter ensured that each individual received an answer sheet with the correct ID number. Answer sheets contained a description of the experiment, in particular the choice set and related consequences for the payoff, and space to write down the decision. Instructions were read aloud by the experimenter. Individuals did not write down their decisions before all clarifying questions had been answered. The subsequent experiments followed the same procedure and were standardized. For a detailed description of each experiment, see Supplementary Material.

To choose the experiment which was relevant for payoff, the teacher was asked to open an envelope which contained a card stating the number of the experiment. The payoff of each individual in the corresponding experiment was calculated and converted into Euro. The exchange rate rate was 75 tokens = 1.00 Euro. Individuals received an additional participation fee of 2.00 Euro per session. Payoffs were handed out to each individual in sealed envelopes by the teacher in exchange for the ID card. We ensured that the maximum possible payoff per session was 10.00 Euro which corresponds to approximately 50% of the recommended monthly amount of pocket money for children aged 11–12 years in Germany (Langmeyer and Winklhofer, 2014). The average individual payoff per session was 6.27 Euro.

## Statistical Analysis

The primary interest of this research is on differences in decisions between the treatment and control group in various economic experiments. As data gathered in economic experiments are rarely normally distributed, we rely on non-parametric tests in the first place. The appropriate-ness of a hypothesis test depends on the level of measurement of our outcome variables (Stevens, 1946). When dealing with categorical or binary data, a χ^2^-test for independence is applied, whereas the Mann-Whitney *U*-test is the choice for ratio data. As hypothesis tests only draw an incomplete picture and have been subject to severe criticism recently (e.g., Büsch and Strauß, 2016; Calin-Jageman and Cumming, 2019), we additionally calculate effect sizes (Cramér's *V* and the correlation coefficient *r*; see Tomczak and Tomczak, 2014) and the corresponding confidence intervals to provide insights on the magnitude of the actual differences between the treatment and the control group.

Beyond plain comparisons between the treatment and control group, we are interested in the causal effect of the intervention on decisions in the economic experiments. The panel structure of the data set allows to address this question using regression analyses. As repeated measurements are not independent, a random effects-model is employed. This allows to control for time-constant independent variables such as gender and socio-economic status (Andreß et al., 2013). To account for correlation within classes, standard errors are clustered.

## Summary

The aim of the study design is to investigate the impact of physical activity and sports participation on social skills using purposefully designed physical education lessons. To test for intervention effects, we utilized a novel approach in this field by applying various incentivized standard economic experiments. To contribute to the existing literature, we designed the *Movigen* (Latin: motio—motion; genitor—creator) project to address two research questions:

1. Are different levels of physical activity associated with different outcomes regarding social skills?
2. Is the intervention using a novel concept for physical education effective in enhancing social skills?

The first question is motivated by the discussion whether sports participation can be considered as investment good. The rationale is that sports participation may not just invoke pleasure but also foster skills which are assumed to translate into favorable outcomes in other domains of life, e.g., success in the labor market (Leeds, 2015). In the present study, this issue can be addressed with data from the first measurement, i.e., before the beginning of the intervention in the treatment group by a comparison of differences in individuals' decisions in the economic experiments contingent on their levels of physical activity. This approach is, however, not appropriate to draw causal conclusions on the relation between the intensity of sports participation and social skills as factors which may affect both domains simultaneously, e.g., due to selection effects, are not explicitly incorporated.

Having a treatment and control group, however, does allow to examine the causal effect of the intervention on decisions in the economic experiments. Due to the repeated measurements in both groups, we are able to disentangle the effect of the intervention on individuals in the treatment group from experience effects which occur if the same series of economics experiments is repeated. Moreover, our design employs an active approach of learning and encourages individuals to develop skills which enable them to solve challenges in various domains of life on their own. This method is expected to be more effective in inducing changes in behavior as compared to approaches which already provide the solution to attain the desired outcome (Durlak et al., 2010). To our best knowledge, there are no previous studies using a comparable approach to test for spillover effects of a sports-related intervention on other domains of life.

## Data Availability Statement

The original contributions presented in the study are included in the article/Supplementary Material, further inquiries can be directed to the corresponding author/s.

## Ethics Statement

The studies involving human participants were reviewed and approved by the Board of Ethics of the Karlsruhe Institute of Technology. Written informed consent to participate in this study was provided by the participants' legal guardian/next of kin.

## Author Contributions

AW and PN conceived the original idea. PN and AH designed the measurements. HW and AW designed the intervention. RW coordinated its implementation. AH drafted the manuscript. RW and HW revised this draft. All authors contributed to the article and approved the submitted version.

## Conflict of Interest

The authors declare that the research was conducted in the absence of any commercial or financial relationships that could be construed as a potential conflict of interest.

## Footnotes

^1^Falk, A., Kosse, F., and Schildberg-Hörisch, H. (2015). *Reducing Overconfidence*. Unpublished manuscript.
