# Supplementary material for: Utilizing Incentivized Economic Experiments to Test for Social Skills Acquisition Through Physical Education: Study Protocol of the Movigen Project
Source: Front Sports Act Living. 2021 May 3;3:587764. doi: 10.3389/fspor.2021.587764 (PMC8126632; doi:10.3389/fspor.2021.587764)
Supplement: Supplementary file 1 [file Data_Sheet_1.PDF]

# Utilizing Incentivized Economic Experiments to Test for Social Skills Acquisition through Physical Education: Study Protocol of the *Movigen* Project

–Supplementary Material–

André Haas[1], Rita Wittelsberger[1], Hagen Wäsche[1], Alexander Woll[1], and Petra Nieken[2]

[1] *Institute of Sports and Sports Science, Karlsruhe Institute of Technology, Karlsruhe, Germany*
[2] *Institute of Management, Karlsruhe Institute of Technology, Karlsruhe, Germany*

July 2020

## Experimental instructions

### General instructions

- Dear participants, welcome to this session. My name is <name of experimenter 1> and together with my colleague <name of experimenter 2> and your teacher <name of teacher> I will guide you through the following 90 minutes.
- Today we want to play different games with you and the other children of your class. For your participation you will receive a payment, depending on the decisions you and the other children of your class will make.
- In each game, you will make decisions which either concern just yourself or other children of your class. The decision you will have to make will be explained in detail at the beginning of each game. In any case, you will receive a minimum amount of 2.00 Euro.
- At the end of the 90 minutes we will pay out one of the games. That means, we will convert all tokens you received in that game into Euro and pay them out in addition to the minimum amount. Therefore, it is worth it to think carefully about your decision in every game.
- The number of the game we will pay out is in this sealed envelope. After we have played the last game, we will ask your teacher to open this envelope.
- Your decisions are confidential, that means neither the other children of your class nor your teacher will learn about your decisions. You will not learn about the decisions of the

other children of your class either. Therefore, it is important that you do not talk about your decisions with the person sitting next to you.

- The payment at the end of the games is also confidential. That means the other children of your class will not learn which payment you receive. You will not learn anything about the payments of the other children of your class either.
- In every game, we use the currency token. At the end, tokens will be converted into Euro. 75 tokens equal 1.00 Euro. Your teacher will do the payment. As stated before, you will receive a minimum amount of 2.00 Euro for your participation.
- At your table you find a card with a number. This number is important in order to ensure that you will receive the correct number of tokens in Euro. Please check on every sheet you receive that the number in the right upper corner corresponds to the number of your card. Only if the numbers are the same we are able to identify your sheet and make sure you receive your payment.
- Please keep this card until you have received your payment. After the last game we will put your payment in an envelope marked with your number. Your teacher will give you this envelope in exchange for the card with your number.
- Before starting a game, we will hand out a new sheet to you. On this sheet you will find a description of the game which we will also read out aloud. If you have any questions, please raise your hand.
- Do you have any questions? If not, we will begin.

**Game 1**

In this game you play together with another child of your class who will be selected randomly after all sheets have been collected.

In this game two players labeled “player 1” and “player 2” play together. You will slip into both roles and make decisions. The other child of your class you will be randomly matched with will do the same. Later on, we will randomly determine whether we take your decision as player 1 or your decision as player 2 into account.

Player 1 receives 600 tokens. She/he can share this amount with player 2 who does not receive any tokens. That is, player 1 makes an offer to player 2 about the allocation of the 600 tokens. Player 2 can decide whether or not she/he wants to accept this offer. If player 2 does not accept, either player will receive no payoff. That means, if player 2 says “no”, both of you receive zero tokens.

First, you make a decision in the role of player 1. You receive 600 tokens and can decide about the offer you want to make to player 2. Player 2 can accept the offer or not. If she/he does not accept, both of you receive zero tokens.

After that, you are in the role player 2 and decide for every possible offer you can get from player 1 whether or not you want to accept. If you do not accept the offer, both of you receive zero tokens.

After you have made your decisions as player 1 and player 2, we determine randomly whether you are player 1 or player 2.

Do you have any questions? If not, we will continue.

Please choose exactly one offer out of the following list you (player 1) want to make to player 2.

- ☐ 600 tokens for player 1 (you) and 0 tokens for player 2
- ☐ 480 tokens for player 1 (you) and 120 tokens for player 2
- ☐ 300 tokens for player 1 (you) and 300 tokens for player 2
- ☐ 120 tokens for player 1 (you) and 480 tokens for player 2
- ☐ 0 tokens for player 1 (you) and 600 tokens for player 2

Now you decide in the role of player 2 whether or not you want to accept each of the following offers.

The following table lists all the possible offers you can get from player 1. Please indicate whether or not you want to accept the offer by ticking the corresponding box. In each line you can tick one box. If you do not accept, both of you receive zero tokens.

Please decide now whether or not you want to accept the following offers and tick the corresponding box.

| Offer | Player 1 | Player 2 (you) | Accept | Not accept |
|---|---|---|---|---|
| 1 | 600 | 0 | ☐ | ☐ |
| 2 | 480 | 120 | ☐ | ☐ |
| 3 | 300 | 300 | ☐ | ☐ |
| 4 | 120 | 480 | ☐ | ☐ |
| 5 | 600 | 600 | ☐ | ☐ |

Please ensure that you ticked one box per line.

**Game 2**

In this game you play together with another child of your class who will be selected randomly after all sheets have been collected.

As in the game before, you will make two decisions. The other child of your class you will be randomly matched with will do the same. First, you choose one color—blue or orange. Second, you make a decision contingent on the choice of a color by the other child of your class.

At the end we will determine randomly which decision of you and and the other child of your class will be realized for the payment. If your first decision will be selected, the second decision of the other child of your class will be realized—and vice versa.

- If you choose the color blue and the other child of your class chooses the color blue, you receive 400 tokens and the other child of your class receives 400 tokens.
- If you choose the color blue and the other child of your class chooses the color orange, you receive 100 tokens and the other child of your class receives 600 tokens.
- If you choose the color orange and the other child of your class chooses the color blue, you receive 600 tokens and the other child of your class receives 100 tokens.
- If you choose the color orange and the other child of your class chooses the color orange, you receive 200 tokens and the other child of your class receives 200 tokens.

First decision: Please decide whether you want to choose the color blue or the color orange.

Second decision: Please decide whether you want to choose the color blue or the color orange if the other child of your class has already chosen blue (orange).

After you have made your decision we determine randomly which decision will be realized for you.

Do you have any questions? If not, we will continue.

We begin with the first decision:

Which color do you choose? Please tick one box.

☐ Blue

☐ Orange

We continue with the second decision:

- Which color do you choose if the other child of your class chooses the color blue? Please tick one box.

  ☐ Blue

  ☐ Orange

- Which color do you choose if the other child of your class chooses the color orange? Please tick one box.

  ☐ Blue

  ☐ Orange

Please ensure that you have ticked one box for every decision.

**Game 3**

In this game you play together with three other children of your class who will be selected randomly after all sheets have been collected.

You and the other children of your class receive 120 tokens. You can allocate these 120 tokens to your own private account or to a collective account in intervals of 24 tokens. For every token you allocate to your own account, you receive 2 tokens in return.

For every token which is allocated to the collective account, you receive 1 token. At the end, we will sum up the total number of tokens which have been allocated to the collective account and distribute this amount to each player. Since everyone can contribute to the shared account, you benefit if other children of your group allocate tokens to the collective account and they benefit from your allocation.

Do you have any questions? If not, we will continue.

How many tokens do you want to allocate to your own account and how many tokens do you want to allocate to the collective account?

In the following illustration, one coin is equal to 24 tokens. Please draw the number of coins you want to allocate to your own account on the left side of the diagram. Likewise, you can draw the number of coins you want to allocate to the collective account on the right side of the diagram.

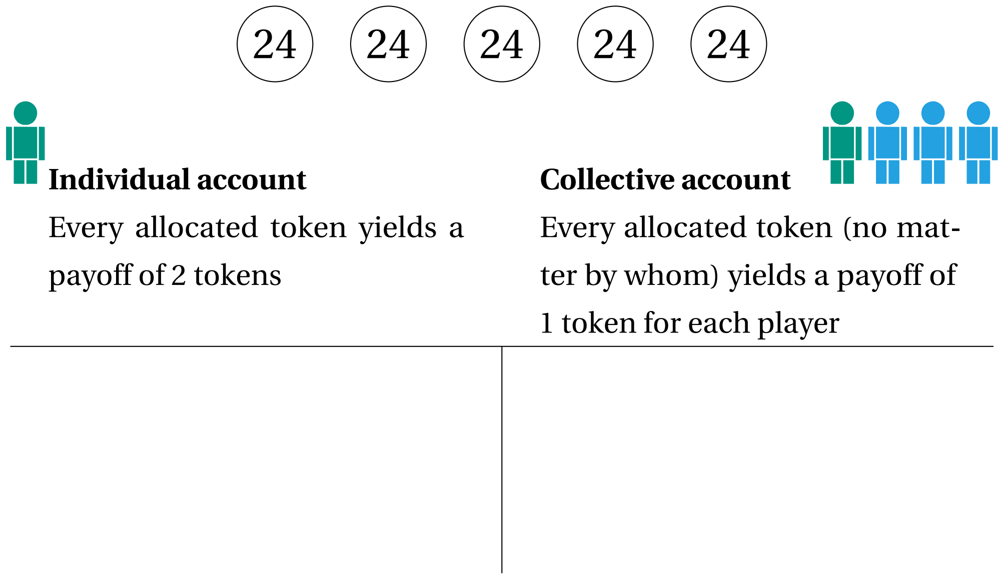


Please ensure that you allocated exactly 5 coins.

**Game 4**

Please do not turn the sheet until we made the start signal.

In this game you can encrypt words into a secret code. To encrypt a word, its letters have to be exchanged by other letters in two subsequent steps. To give you an idea how it works, consider the following example.

The tables below show the replacement for each letter:

1st step

| Replace | A | B | C | D | E | F | G | H | I | J | K | L | M | N | O | P | Q | R | S | T | U | V | W | X | Y | Z |
|---|---|---|---|---|---|---|---|---|---|---|---|---|---|---|---|---|---|---|---|---|---|---|---|---|---|---|
| with | K | L | W | U | B | F | N | M | O | E | P | J | A | I | S | D | V | X | R | G | Y | H | Z | C | T | Q |

2nd step

| Replace | A | B | C | D | E | F | G | H | I | J | K | L | M | N | O | P | Q | R | S | T | U | V | W | X | Y | Z |
|---|---|---|---|---|---|---|---|---|---|---|---|---|---|---|---|---|---|---|---|---|---|---|---|---|---|---|
| with | P | T | F | E | S | D | U | O | G | V | B | Z | H | N | M | C | R | Q | Y | I | X | W | J | K | L | A |

In the following example you are asked to encrypt the given word by subsequently replacing its letters with letters from the first and the second table, respectively. This example helps you to find out whether you find the task easy or difficult. You have 30 seconds to work on the example.

| S | A | L | T | O |
|---|---|---|---|---|
| | | | | |

On the back of this sheet we have added more words you can encrypt into the secret code. You have 2 minutes to encrypt as many words as possible. For every correctly encrypted letter you receive 3 tokens with a maximum of 300 tokens.

Before we start, we want you to give an estimate how well you perform in comparison to the other children of your class. If you take all children of your class into account, do you expect yourself to be among the upper, the middle, or the lower third? If your estimation is correct you receive 300 tokens and zero tokens otherwise.

Do you have any questions? If not, we will continue.

Do you expect yourself to be among the upper, the middle, or the lower third of your class? Please tick one box.

- ☐ Upper third
- ☐ Middle third
- ☐ Lower third

On the back of this sheet you find more words which are to be encrypted into the secret code. Please do not turn the play bar until we made the start signal. After we made the start signal, you have two minutes to encrypt as many words two times as possible. The moment we make the end signal, please cease writing immediately.

**Game 5**

Please do not turn the sheet until we made the start signal.

In this game you can again encrypt words into a secret code. As in the game before, the letters have to be exchanged by other letters in two subsequent steps. The tables that show the replacement for each letter, however, have changed.

You have two minutes to encrypt the words on the back of the sheet. Before we start, you can choose one out of two payoff schemes:

1. For every correctly encoded letter you receive 3 tokens with a maximum of 300 tokens.
2. We compare your result with the result of another child of your class who will be selected randomly. The one with the higher number of correctly encoded letters receives 600 tokens. The other one receives zero tokens. In case of a tie we will determine randomly who receives 600 tokens and who receives zero tokens.

Do you have any questions? If not, we will continue.

Which payoff scheme do you choose? Please tick one box.

- ☐ 3 tokens for every correctly encoded letter
- ☐ 600 tokens for you if you encode more letters correctly than the other randomly-selected child of your class and zero tokens otherwise

On the back of this sheet you find more words which are to be encrypted into the secret code. Please do not turn the play bar until we made the start signal. After we made the start signal, you have 2 minutes to encrypt as many words two times as possible. The moment we make the end signal, please cease writing immediately.

**Game 6**

Please do not turn the sheet until we made the start signal.

In this game you can again encrypt words into a secret code. As in the game before, the letters have to be exchanged by other letters. The tables that show the replacement for each letter, however, have changed.

You have two minutes time to encrypt the words on the back of the sheet. Before we start, you can choose one out of three alternatives:

- Alternative 1 requires one-fold encryption. That is, the letters of the original words have to be replaced by the letters in the upper table. If you encrypt at least five letters correctly, you receive 1 token for every correctly encrypted letter with a maximum of 100 tokens. If you encrypt less than five letters correctly, you receive zero tokens.
- Alternative 2 requires two-fold encryption. That is, the letters of the original words have to be replaced first by the letters in the upper table and then by the letters in the middle table. If you encrypt at least 15 letters correctly, you receive 3 tokens for every correctly encrypted letter with a maximum of 300 tokens. If you encrypt less than 15 letters correctly, you receive zero tokens.
- Alternative 3 requires three-fold encryption. That is, the letters of the original words have to be replaced first by the letters in the upper table, then by the letters in the middle table,

and finally by the letters in the lower table. If you encrypt at least 35 letters correctly, you receive 10 tokens for every correctly encrypted letter with a maximum of 1,000 tokens. If you encrypt less than 35 letters correctly, you receive zero tokens.

Do you have any questions? If not, we will continue.

Which alternative do you choose? Please tick one box.

- ☐ Alternative 1 (one-fold encryption): If you encrypt at least five letters correctly, you receive 1 token for every correctly encrypted letter.
- ☐ Alternative 2 (two-fold encryption): If you encrypt at least 15 letters correctly, you receive 3 tokens for every correctly encrypted letter.
- ☐ Alternative 3 (three-fold encryption): If you encrypt at least 35 letters correctly, you receive 10 token for every correctly encrypted letter.

On the back of this sheet you find more words which are to be encrypted into the secret code. Please do not turn the play bar until we made the start signal. After we made the start signal, you have 2 minutes to encrypt as many words two times as possible. The moment we make the end signal, please cease writing immediately.

**Game 7**

In this game you can choose between two envelopes that are labeled envelope A and envelope B.

Envelope A contains 6 blue cards. Envelope B contains 3 red and 3 green cards.

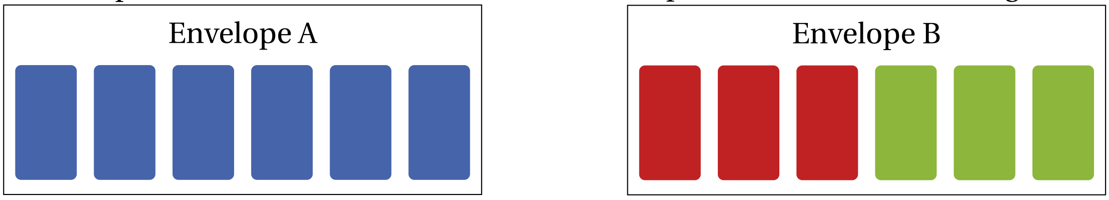


Your payoff depends on color of the card which will be drawn randomly. If this game is selected for payment at the end of the session, we will ask your teacher to draw one card randomly from each envelope.

- If a blue card is drawn, you receive 200 tokens.
- If a red card is drawn, you receive 600 tokens.
- If a green card is drawn, you receive 0 tokens.

Do you have any questions? If not, we will continue.

Which envelope do you choose? Please tick one box.

- ☐ Envelope A
- ☐ Envelope B

**Game 8**

In this game there are two other envelopes which are labeled envelope C and envelope D. Again, you can choose one of the two envelopes.

Envelope C contains 6 orange cards.

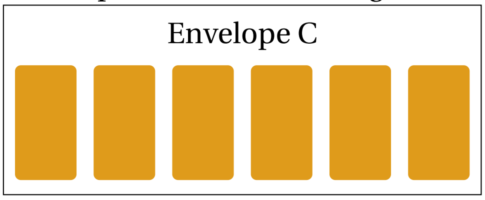


Envelope D contains 3 purple and 3 yellow cards.

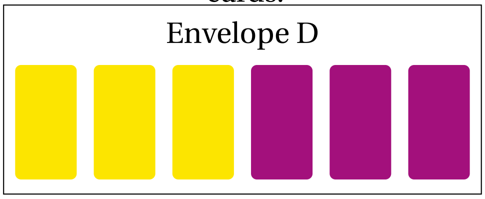


Your payoff depends on color of the card which will be drawn randomly. If this game is selected for payment at the end of the session, we will ask your teacher to draw one card randomly from each envelope.

- If an orange card is drawn, you receive 400 tokens.
- If a purple card is drawn, you receive 600 token.
- If a yellow card is drawn, you receive 0 tokens.

Do you have any questions? If not, we will continue.

Which envelope do you choose? Please tick one box.

☐ Envelope C

☐ Envelope D

**Game 9**

At the beginning of this game you receive 600 tokens. You can keep these 600 tokens or pass a fraction thereof to the relief organization *Save the Children*. Your decision will not be disclosed to anyone in this class, i.e., neither your teacher nor the other children of your class will learn about your decision.

Around the world, *Save the Children* fights for children's rights and helps them fulfill their potential. They provide lifesaving supplies and emotional support for children caught up in disasters like floods, famine, and wars. In the long run, *Save the Children* wants to ensure that children have access to life-saving healthcare and quality education.

If you pass a fraction of your tokens *Save the Children*, we will donate this amount to *Save the Children* if this game is selected for payment at the end of the session.

Do you have any questions? If not, we will continue.

Please choose in the list below how many tokens you want to keep for yourself and how many tokens you want to pass to *Save the Children*. Please tick one box.

☐ Keep 600 tokens and pass 0 tokens to *Save the Children*

☐ Keep 480 tokens and pass 120 tokens to *Save the Children*

☐ Keep 300 tokens and pass 300 tokens to *Save the Children*

☐ Keep 120 tokens and pass 480 tokens to *Save the Children*

☐ Keep 0 tokens and pass 600 tokens to *Save the Children*

**Game 10**

Please imagine a number between 1 and 6.

Now you receive an envelope. This envelope contains six cards with numbers from 1 to 6.

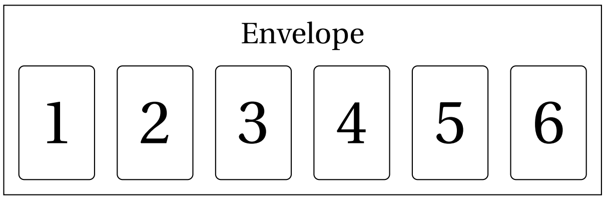


Please draw one card from the envelope. Have a look at the card and put the card back into the envelope. If the number on the card is the same as the number you have imagined, you receive 600 tokens. If the number on the card is different from the number you have imagined, you receive zero tokens.

Do you have any questions? If not, we will continue.

Is the number on the card you have drawn the same as the number you have imagined? Please tick one box.

☐ Yes

☐ No

Please ensure that you checked one box.

**Game 11**

In this game you can choose between two payoffs. You either receive 300 tokens today or you receive 600 tokens in one week.

Do you have any questions? If not, we will continue.

Which payoff do you choose? Please tick one box.

☐ 300 tokens today

☐ 600 tokens in one week

Please ensure that you checked one box.
